# Association between in vitro susceptibility and clinical outcomes in fungal keratitis

**DOI:** 10.1186/s12348-024-00418-w

**Published:** 2024-09-02

**Authors:** Louisa Lu, N. Venkatesh Prajna, Prajna Lalitha, Revathi Rajaraman, Muthiah Srinivasan, Benjamin F. Arnold, Nisha Acharya, Thomas Lietman, Jennifer Rose-Nussbaumer

**Affiliations:** 1 Department of Ophthalmology, Byers Eye Institute, Stanford University, 2542 Watson Ct, Palo Alto, Stanford, CA 94303 USA; 2 Aravind Eye Hospital, Coimbatore, Tamil Nadu India; 3 Francis I. Proctor Foundation, University of California, San Francisco, San Francisco, CA USA; 4 Department of Ophthalmology, University of California, San Francisco, San Francisco, CA USA; 5 Department of Epidemiology and Biostatistics, University of California, San Francisco, San Francisco, CA USA

**Keywords:** Fungal keratitis, Microbial susceptibility, Antifungals, Minimum inhibitory concentration, Therapeutic penetrating keratoplasty

## Abstract

**Purpose:**

The purpose of this study was to assess the association between antifungal susceptibility as measured by minimum inhibitory concentration (MIC) and clinical outcomes in fungal keratitis.

**Methods:**

This pre-specified secondary analysis of the Mycotic Ulcer Treatment Trial II (MUTT II) involved patients with filamentous fungal keratitis presenting to Aravind Eye Hospitals in South India. Antifungal susceptibility testing for natamycin and voriconazole was performed on all samples with positive fungal culture results according to Clinical and Laboratory Standards Institute Guidelines. The relationship between MIC and clinical outcomes of best-corrected visual acuity, infiltrate or scar size, corneal perforation, need for therapeutic penetrating keratoplasty, and time to re-epithelialization were assessed.

**Results:**

We obtained MIC values from 141 patients with fungal keratitis. The most commonly cultured organisms were Aspergillus (46.81%, *n* = 66) and Fusarium (44.68%, *n* = 63) species. Overall, there was no association between antifungal MICs and clinical outcomes. Subgroup analysis revealed that among *Fusarium*-positive cases, higher voriconazole MIC was correlated with worse three-month best-corrected visual acuity (*p* = 0.03), increased need for therapeutic penetrating keratoplasty (*p* = 0.04), and time to re-epithelialization (*p* = 0.03). No significant correlations were found among *Aspergillus*-positive cases. There were no significant correlations found between natamycin MIC and clinical outcomes among organism subgroups.

**Conclusions:**

Decreased susceptibility to voriconazole was associated with increased odds of requiring a therapeutic penetrating keratoplasty in *Fusarium*-positive cases. Susceptibility to natamycin was not associated with any of the measured outcomes.

## Background

Fungal keratitis affects over a million people annually and causes significant morbidity and blindness worldwide. Management involves obtaining diagnostic corneal scrapings for culture and timely initiation of empiric topical antifungal agents. Results from studies in the literature have suggested that clinical outcomes of fungal keratitis may be correlated with the in vitro susceptibility of cultured isolates to antifungals. This study aimed to explore the role of susceptibility testing in guiding the management of fungal keratitis by assessing the association between antifungal susceptibility as measured by minimum inhibitory concentration (MIC) and clinical outcomes such as best-corrected visual acuity, infiltrate or scar size, corneal perforation and/or therapeutic penetrating keratoplasty, and time to re-epithelialization.

## Introduction

Results from studies in the literature have suggested that clinical outcomes of fungal keratitis may be correlated with the in vitro susceptibility of cultured isolates to antifungals, but the role of susceptibility testing in guiding the management of fungal keratitis remains undetermined [1–6].

Fungal keratitis affects over a million people annually and causes significant morbidity and blindness worldwide, particularly among young, male agriculture workers of low socioeconomic status in regions with tropical and subtropical climates [7–11]. Management involves obtaining diagnostic corneal scrapings for culture and timely initiation of empiric topical antifungal agents, with topical natamycin being the preferred first-line agent against filamentous fungal keratitis [12].

The Mycotic Ulcer Treatment Trial II (MUTT II) was a randomized clinical trial that did not find a benefit to adding oral voriconazole to topical antifungal agents in the treatment of severe filamentous fungal keratitis [13]. In this secondary analysis of the MUTT II, we investigate the association between in vitro antifungal susceptibility–as measured by minimum inhibitory concentration (MIC)–and clinical outcomes, including best-corrected visual acuity, infiltrate or scar size, corneal perforation and/or therapeutic penetrating keratoplasty, and time to re-epithelialization, during the course of treatment.

## Methods

The MUTT II was a multicenter, double-masked, placebo-controlled randomized clinical trial that compared clinical outcomes in study participants with severe fungal corneal ulcers and were being treated with topical antifungals who received adjuvant oral voriconazole versus placebo. The methods for MUTT II have been previously reported in detail [13]. In summary, patients with culture-positive filamentous fungal corneal ulcers and a baseline visual acuity of 20/400 or worse were randomized to receive oral voriconazole or a placebo. All study participants received topical voriconazole, 1%, and after the results of MUTT I were available, topical natamycin, 5%, was also administered.

Corneal scrapings and cultures were obtained from all patients for fungal cultures at time of enrollment in the study. Fungal cultures were determined to be positive if there was growth on two or more media or moderate to heavy growth on one medium, and fungal identification was performed using gross and microscopic characteristics. All specimens with positive fungal culture results underwent antifungal susceptibility testing for natamycin and voriconazole using broth microdilution according to standardized methods described in the Clinical and Laboratory Standards Institute document M38-A2 [14]. The Minimal Inhibitory Concentrations (MICs) for natamycin and voriconazole were recorded as the lowest concentration of the antifungal that inhibited growth of the organism, observed as a 100% visual reduction in turbidity when compared with the control tube for natamycin at 48 h, and an 80% reduction in turbidity for voriconazole.

A log2 transformation of MIC was used for all statistical models. The relationship between MIC and clinical outcomes of best-corrected visual acuity, infiltrate or scar size, corneal perforation and/or therapeutic penetrating keratoplasty, and time to re-epithelialization was analyzed by multivariable logistic regression modeling for dichotomous outcomes and multivariable linear regression modeling for continuous outcomes. All statistical analyses were performed using R Statistical Software (version 4.3.0, R Foundation for Statistical Computing, Vienna, Austria).

## Results

Of the 240 patients enrolled in the clinical trial, 141 (59%) had available data on fungal culture speciation and MIC data for natamycin and voriconazole and were included in the analysis. Baseline clinical characteristics for participants included in this study are described in Table 1.

**Table 1:** Clinical characteristics of participants with available culture speciation

Characteristics (*n* = 141)	
Sex, No. (%)	
Male	80 (56.64)
Female	61 (43.26)
Age, median in years	52
Baseline visual acuity (LogMAR), median (IQR)	1.7 (1.5–1.8)
Baseline infiltrate and/or scar size (mm), median (IQR)	5.49 (4.58–6.54)
Experienced corneal perforation, No. (%)	41 (29.08)
Time to re-epithelialization (days), median (IQR)	20 (9–21)

The most commonly cultured organisms were Aspergillus (47%, *n* = 66) and Fusarium (45%, *n* = 63) species. The MICs of natamycin and voriconazole for the cultured species are noted in Table 2.

**Table 2:** Minimum inhibitory concentration (MIC) for natamycin and voriconazole

		Natamycin		Voriconazole	
Organism	Count (% of total)	Minimum Inhibitory Concentration (μg/mL)		Minimum Inhibitory Concentration (μg/mL)	
		Median MIC	MIC Range	Median MIC	MIC Range
*Fusarium* species	63 (45%)	4	2–32	4	0.25-16
*Aspergillus* species	66 (47%)	32	2–64	0.5	0.25-25
*A. flavus*	53 (38%)	32	2–64	0.5	0.25-25
*A. fumigatus*	2 (1%)	3	2–4	8.25	0.5–16
* A. terreus*	2 (1%)	12	8–16	0.5	0.5
*Alternaria*	1 (1%)	1	1	2	2
*Bipolaris*	1 (1%)	2	2	1	1
*Collecotrichum species*	1 (1%)	2	2	0.5	0.5
*Exerohilum* species	1 (1%)	2	2	1	1
*Lasiodiplodia* species	2 (1%)	2	2	2.25	0.5-4
*Penicillium* species	1 (1%)	16	16	0.25	0.25
Unidentified dematiaceous	2 (1%)	1.5	1–2	3	2–4
Unidentified hyaline	3 (2%)	2	2–8	1	0.5-2
Total (*n* = 240)	141	8	1–64	1	0.25-25

The association between MIC and clinical outcomes, both overall and among *Fusarium* and *Aspergillus* isolates specifically, are presented in Tables 3 and 4.

**Table 3:** Minimum inhibitory concentration (MIC) predicting visual acuity, scar size, and time to re-epithelialization

Clinical Outcome	Subgroup (*n*)	Natamycin MIC*			Voriconazole MIC*		
		Difference	95% CI	*P*-value	Difference	95% CI	*P*-value
Three-month best-corrected visual acuity, logMAR	All fungal isolates (*n* = 141)	0.01	-0.08-0.10	0.83	0.03	-0.04-0.11	0.46
	*Fusarium* isolates only (*n* = 63)	-0.10	-0.37-0.16	0.44	0.18	0.02–0.34	**0.03**
	*Aspergillus* isolates only (*n* = 66)	0.08	-0.14-0.29	0.38	-0.06	-0.20-0.08	0.49
Three-month infiltrate or scar size, mm	All fungal isolates (*n* = 141)	-0.04	-0.27-0.18	0.71	0.02	-0.16-0.21	0.82
	*Fusarium* isolates only (*n* = 63)	-0.36	-1.08-0.36	0.32	0.07	-0.34-0.48	0.74
	*Aspergillus* isolates only (*n* = 66)	0.11	-0.42-0.65	0.67	-0.20	-0.55-0.15	0.26
Time to re-epithelialization, days	All fungal isolates (*n* = 141)	0.16	-2.48-2.80	0.90	-1.24	-3.33-0.85	0.24
	*Fusarium* isolates only (*n* = 63)	-1.00	-8.42-6.42	0.79	-4.61	-8.66-[-0.55]	**0.03**
	*Aspergillus* isolates only (*n* = 66)	1.05	-5.38-7.48	0.74	2.72	-1.7-7.14	0.22

* Estimated difference and 95% Confidence Interval (CI) associated with a log_2_ increase in MIC

**Table 4:** Minimum inhibitory concentration (MIC) predicting corneal perforation and therapeutic penetrating keratoplasty

Clinical Outcome	Subgroup	Natamycin MIC *			Voriconazole MIC *		
		OR	95% CI	*P*-value	OR	95% CI	*P*-value
Corneal perforation	All fungal isolates (*n* = 141)	1.03	0.76–1.40	0.83	0.99	0.78–1.26	0.96
	*Fusarium* isolates only (*n* = 63)	0.76	0.25–1.98	0.60	1.05	0.62–1.84	0.87
	*Aspergillus* isolates only (*n* = 66)	1.03	0.53–2.16	0.94	0.65	0.29–1.12	0.20
Therapeutic penetrating keratoplasty	All fungal isolates (*n* = 141)	0.89	0.75–1.31	0.92	1.15	0.92–1.44	0.22
	*Fusarium* isolates only (*n* = 63)	0.56	0.19–1.48	0.26	1.92	1.11–3.89	**0.04**
	*Aspergillus* isolates only (*n* = 66)	0.90	0.47–1.69	0.74	0.95	0.60–1.46	0.81

* Estimated Odds Ratio (OR) and 95% Confidence Interval (CI) associated with a log_2_ increase in MIC

Analysis of the combined subgroups involving all cultured isolates revealed no association between antifungal MICs and outcomes of best-corrected visual acuity (natamycin: 95% CI, -0.08-0.10; *P* = 0.83; voriconazole: 95% CI, -0.04-0.11; *P* = 0.46), infiltrate or scar size (natamycin: 95% CI, -0.27-0.18; *P* = 0.71; voriconazole: 95% CI, -0.16-0.21; *P* = 0.82), corneal perforation (natamycin: odds ratio, 1.03; 95% CI, 0.76–1.40; *P* = 0.83; voriconazole: odds ratio, 0.99, 95% CI, 0.78–1.26; *P* = 0.96), need for therapeutic penetrating keratoplasty (natamycin: odds ratio, 1.15; 95% CI, 0.75–1.31; *P* = 0.92; voriconazole: odds ratio, 0.99; 95% CI, 0.92–1.44; *P* = 0.22), and time to re-epithelialization (natamycin: 95% CI, -2.48-2.80, *P* = 0.90; voriconazole: 95% CI, -3.33-0.85, *P* = 0.24).

Subgroup analysis by cultured organism, however, revealed that among *Fusarium*-positive cases, a two-fold increase in voriconazole MIC was significantly correlated with increased odds of needing therapeutic penetrating keratoplasty (odds ratio, 1.92; 95% CI, 1.11–3.89; *P* = 0.04), three-month best-corrected visual acuity (95% CI, 0.02–0.34; *P* = 0.03), and time to re-epithelialization (95% CI, -8.66-[-0.55]; *P* = 0.03). No significant correlations were found among *Aspergillus*-positive cases. Natamycin MIC was not found to be a significant predictor of any of the studied clinical outcomes among organism subgroups.

## Discussion

This study investigated the relationship between in vitro antifungal susceptibility, as measured by minimum inhibitory concentration (MIC), and clinical outcomes among study participants enrolled in MUTT II. We found that decreased susceptibility to voriconazole correlated with worse three-month best-corrected visual acuity, increased odds of undergoing therapeutic penetrating keratoplasty, and increased time to re-epithelialization in *Fusarium-*positive cases. Susceptibility to natamycin was not associated with any of the measured outcomes in the study.

Our findings can be compared to other studies in the literature regarding fungal keratitis and susceptibility, including a previous analysis by our group of in vitro susceptibility and clinical outcomes in the MUTT I clinical trial [1]. Analysis of MIC data among study participants in MUTT I found that decreased susceptibility to natamycin correlated with increased infiltrate or scar size as well as increased odds of corneal perforation, and that susceptibility to voriconazole was not associated significantly with any measured outcomes. Patients in MUTT I had overall less severe clinical presentations than patients in MUTT II, which may partially account for the difference in results among the associations between clinical outcomes and susceptibility to natamycin or voriconazole in MUTT I versus this current study of MUTT II. The patients in MUTT II also received both topical voriconazole as well as topical natamycin once the results of MUTT I were available, which may have caused additional drug-induced corneal toxicity contributing to more severe clinical presentations. Another study of fungal susceptibility testing and clinical outcomes also found a linear correlation between susceptibility and outcome in fungal keratitis [5]. Evaluated together, the results from studies of MIC and clinical outcomes in MUTT I and MUTT II suggest that in vitro antifungal resistance may be associated with worse clinical outcomes in fungal keratitis, including corneal perforation and lack of response to medical therapy, thereby increasing the odds of undergoing a therapeutic penetrating keratoplasty.

Reports of the in vitro activity of natamycin and voriconazole against filamentous fungal isolates from a large sample of fungal keratitis cases in southern India found that *Fusarium* isolates were less susceptible to voriconazole relative to other organisms, and *Aspergillus* isolates had lower susceptibility to natamycin compared to other organisms [2]. While clinical breakpoints, or standardized MIC threshold values, have yet to be set by the Clinical and Laboratory Standards Institute for natamycin, the clinical breakpoint for voriconazole was set for *Aspergillus* as an MIC of 1 μg/mL, and an epidemiologic cutoff value has been proposed for voriconazole against *Fusarium* as an MIC of 4 μg/mL [15, 16]. As most of the *Fusarium* isolates in our study sample were at or above this threshold MIC for voriconazole, the results from our study sample revealed an overall lower susceptibility of *Fusarium* isolates to voriconazole. Given that the majority of isolates may have been resistant strains, the significant correlation between higher voriconazole MIC and specific clinical outcomes in this study suggest a quantitative rationale for previous findings that *Fusarium*-positive corneal ulcers have have poorer clinical outcomes when randomized to treatment with voriconazole versus natamycin [12, 17].

Other studies have also reported susceptibility trends among *Fusarium* and *Aspergillus* isolates to different classes of antifungal drugs over the past decade [18–22]. Changing susceptibility patterns among fungal isolates highlight the notion that in vitro susceptibility testing may be useful in guiding treatment decisions in fungal keratitis.

In conclusion, this study investigated the association between antifungal susceptibility as measured by minimum inhibitory concentration (MIC) and clinical outcomes in fungal keratitis cases in South India that were treated with natamycin and voriconazole. Decreased susceptibility to voriconazole was associated with increased odds of requiring a therapeutic penetrating keratoplasty in *Fusarium*-positive cases. Susceptibility to natamycin was not associated with any of the measured outcomes. Microbiological susceptibility results may be useful for risk stratification of patients with severe fungal keratitis to identify patients with the highest likelihood of developing poor clinical outcomes, including corneal perforation and need for therapeutic penetrating keratoplasty.

## Data Availability

The datasets used and/or analyzed during the current study are available from the corresponding author on reasonable request.

## Abbreviations

MIC: Minimum inhibitory concentration
MUTT: Mycotic Ulcer Treatment Trial
